# The Hospital. Nursing Section

**Published:** 1903-01-03

**Authors:** 


### The Hospital.

### Nursing Section.

Contributions for this Section of "The Hospital" should be addressed to the Editor, "The Hospital" Nursing Section, 28 & 29 Southampton Street, Strand, London, W.C.

No. 849.—Vol. XXXIII. *SATURDAY, JANUARY* 3, 1903.

### Notes on News from the Nursing World.

### ROYAL NATIONAL PENSION FUND FOR NURSES.

The record year of the Royal National Pension Fund for Nurses has been beaten, and striking as the figures were for 1901, those for 1902 are still more satisfactory. The new policies issued during the year amounted to upwards of 860; the premiums received exceeded £93,000; the pensions paid were over £7,500; and the sick pay distributed over £1,550. The invested funds now exceed £725,000 sterling; over £13,000 has been distributed in sick pay since the Fund was established, and more than £125,000 has been returned to nurses who have been obliged to give up their policies. During the year her Majesty receives the nurses the Fund always has a large accession to its members, but in 1902 there was no reception to account for the better results. The increase in the premiums paid is of all features the most encouraging. The amount distributed last year in pensions was £1,600 in excess of 1901; this alone represents a capital of £40,000. Moreover, each year the harvest of thrift will be larger and larger, and thus afford ever-increasing evidence of the great value of the organisation to nurses. That they appreciate it more and more each year is conclusively shown by the steady growth in the number of policies taken out.

### THE JUNIUS S. MORGAN BENEVOLENT FUND.

The Benevolent Fund of the Nurses' Pension Fund has just received the largest legacy which has yet come its way. The late Mr. Francis T. Freeman left a considerable sum of money to be distributed at the discretion of the executors of his will, and largely through the advocacy of Mr. Louis Dick, the executors have given £1,000 to the Benevolent Fund. It must be remembered that the Benevolent Fund has never yet made any public appeal for money, and has been almost entirely maintained, as well as established, by the Junius S. Morgan family, by nurses and their friends. Some few generous gifts and legacies have come quite unsolicited from outside sources, and have been most welcome, as the Benevolent Fund has much to do to help those nurses of the Pension Fund who meet with misfortune. In the past year two legacies and one donation were received, and we hope that 1903 will bring a further number of gifts.

### NURSES AT THE QUEEN'S DINNER.

By the express desire of the Queen, Dr. J. J. Marsh, surgeon of the Belgravia nursing division of the St. John's Ambulance Brigade, assisted by Colonel J. Lees Hall, R.A.M.C., was in attendance with a staff of five emergency ambulance nurses at the Christmas dinner given by her Majesty on Saturday to the widows and orphans of soldiers and sailors who were killed in the South African War. Happily, however, the proceedings passed off without their services being seriously in demand, and the nurses had the gratification of witnessing a bright and memorable spectacle.

### PLAGUE NURSES AND HONOURS.

It is unlikely that the splendid work done by nurses attending patients suffering from plague in India and elsewhere will be overlooked by the authorities. For those who died at their post there was only possible the reward that life itself had been well laid down in the performance of duty. But for those who have passed unscathed through the ordeal of exposure to the disease, or who have recovered from an attack, there is, we assume, as much certainty of recognition at the hands of the State as there was in the case of the nurses who ministered with such success and self-denial to our sick and wounded soldiers in South Africa.

### THE REPORT OF THE DEPARTMENTAL COMMITTEE.

At the annual meeting of the Manchester and District Poor Law Officers' Association, Mr. R. A. Leach, the president, said that the recommendations contained in the report of the departmental committee on nursing "would receive the early attention of the district executive," but added that he approved of the proposals of the committee, though he was not prepared to commit himself to details. We notice in this connection that the Guardians of Richmond, Surrey, have anticipated the order which they think the Local Government Board will issue, adopting the recommendations of the committee, and effusively appointed as "qualified nurses" eight young women who have had a year's training.

### THE INDEFINITE POSITION OF POOR LAW NURSES.

The reports on Poor Law administration for 1901 and 1902 contain, as usual, some interesting remarks by the inspectors on nursing in workhouse infirmaries. Mr. Hervey, the inspector for the Eastern counties, says that "the condition of nursing is in need of improvement," and the position of the nurses in workhouses requires defining. "At present," he adds, "owing to a certain indefiniteness, there is in places a good deal of friction which might and should be impossible." Mr. Hervey does not enlarge on "the difficulties in obtaining and keeping satisfactory nurses in the country workhouses, nor on the various means by which a sufficient and continual supply might be obtained," because the question is now receiving much attention from the Local Government Board.

### THE CLASSIFICATION PROPOSAL.

MR. FLEMING finds that in his district, comprising Dorset, Hants, the Isle of Wight, Farnham, and Wilts, nursing has continued the advance referred to last year, while difficulties have diminished as efficiency has progressed. The staff at Portsmouth Parish Infirmary, he states, has been further increased, and the training is progressing quite smoothly, and the Farnham guardians are placing their nursing arrangements on a permanent and satisfactory footing. They promise to be so good that the infirmary, we learn with pleasure, is likely to become a valuable training school for probationers. Mr. Fleming criticises the proposal that the sick should be classified into one or two workhouses in each county, and the assertion that by this means the nursing arrangements might be made so much better that they might be free from the objections which are alleged to attach to workhouse nursing, and that, in consequence, the difficulties in obtaining good candidates would be removed. He observes, with obvious cogency, that the nursing ought to be adapted to the requirements of the patients, not that the interests of the patients should be subservient to the convenience of the nursing arrangements; and he contends that such a scheme could not be carried out without great hardship to the poor. The patients, he pleads, should not be subjected to the pang of removal from the workhouse among their own people to the suggested infirmary among strangers, and he urges that "the fretting and regret for local associations would more than undo the benefit which might be hoped for from arrangements which in theory would be more complete, but which in fact would leave aside the personal considerations and the quiet mind which are among the most important factors in obtaining favourable conditions for the treatment of the sick."

### THE RELIGIOUS QUESTION AT GLASGOW.

IT seems that Edinburgh is not the only important city in Scotland where the religious question is causing agitation among nurses and their friends. A candidate for a probationership at one of the Glasgow hospitals applied for admission and promptly received an application form, which she as promptly filled up. One of the printed questions is, "What is applicant's religion?" In this case the answer was, "Roman Catholic." The candidate had a reply to her application, almost by return of post, to the effect that "any vacancies were filled up for over two years." Not unnaturally, she suspects that her religion blocked the way, and, in any event, if it were known when she originally wrote that there could not be any vacancies for over two years, what was the use of sending the form and putting the candidate to the trouble of obtaining testimonials? It is of the first importance that the governing bodies of great charitable institutions in Scotland, suspected of a desire to exclude Roman Catholics from the nursing staff, should take steps to make it clear that they do not permit the imposition of a religious test.

### DEATH OF A SISTER ON CHRISTMAS EVE.

THE Christmas season at Jessop Hospital for Women at Sheffield was overshadowed by the death, on Christmas Eve, of Miss Elizabeth Hickman, Sister in charge of the theatre for ten years, from gastric fever. The body was subsequently removed to Birmingham for interment, the funeral arrangements being provided for by the Hospital Board. The chairman, Colonel Cutler, was present during the removal. Wreaths were sent by the medical staff, the matron, and the nurses of Jessop Hospital, and also by the domestic staff and patients, among whom Sister Hickman was very popular. Her services were also much appreciated by the hospital authorities.

### ABOLISHING INCOMPETENT MATERNITY "NURSES."

A VERY important decision was unanimously arrived at by the members of the Epsom District Nursing Association at their fifteenth annual meeting. Dr. Beaumont, having dwelt at length in his speech on the need of getting rid of incompetent maternity nurses in the district, proposed that three fully-trained nurses attached to the association should be subsidised so as to render them liable to be called upon by the doctors, the district visitors or the clergy, to attend cases among the poor. This proposal, with a strong expression of opinion in favour of no fees, was adopted, the chairman, Dr. Coltart, remarking that he thought they had thus initiated a splendid work in Epsom. The new departure is the more justifiable and opportune at the present moment because the association has a substantial balance in hand and can well afford to pay the nurses an extra sum for attendance in maternity cases. If the policy determined upon at Epsom is generally carried out by district nursing organisations in the country, the incompetent maternity "nurse" will soon become a creature of the past.

### DUTCH PROBATIONERS IN BURGHER CAMPS.

THE refugee camps in South Africa will soon be a thing of the past, but it will be a long time before English sisters forget their experiences of Dutch probationers. A sister now at Klerksdorp states that they candidly said they came because they wanted to earn a little money, and when opportunity offered they would give up nursing and return to their homes. Their notions of law and order were more than weird. One morning when the doctor went his round no trace of the two damsels left on duty could be found. Finally, they were discovered, one reposing peacefully on an empty bed, wrapped in slumber, while the other had been shampooing her hair, and was processing about with it hanging down her back to dry. Another probationer, during her first day on trial, was filled with unrighteous indignation because she was not allowed to cleanse five infants in the same pint of exceedingly opaque water and to use the swab with which she had previously washed the teacups. The following morning she sent up a message to the effect that hospital work was much too hard for her. Some of the probationers went in for amateur doctoring, one very self-confident young woman applying mustard leaves freely, for any and every description of pain when she had the chance, while another of the "know-all" type proceeded to beautify the eyes of several male patients by dropping in atropine, because she had seen the sister using it for a special case in the ophthalmic ward. A very new recruit was found by the night sister washing her pedal extremities while on duty in the ward, and when remonstrated with cheerfully remarked that the elders and betters, including the doctor, were asleep and it was "all right." On the other hand, many of the Dutch probationers were extremely helpful and most willing. They were very punctual, turning up on duty before their time, and, as a rule, they took rebuke in very good part. The threat of reporting them to the matron was generally quite sufficient, for they had a genuine regard and esteem for her and wished to keep in her good books.

### THE NECESSITY OF CORRECT CERTIFICATES.

IT is reasonable to expect that the certificate of a nurse's training represents exactly what it says. Miss Dillwyn was therefore more than justified in the protest she made at a meeting of the governing body of Swansea Hospital against a certificate of three years' training being presented to her for signature, when, as a matter of fact, the third year of the probationer had been spent in private nursing. At the time Miss Dillwyn attached her signature to the document she was not aware of this circumstance. We do not know who is to blame in the case, but it is obvious that all certificates should be verbally and even literally accurate, and that a member of a committee who is officially asked to append his signature to any certificate which implies what is not true has a serious cause of complaint.

### GOOD WORK AT CHELTENHAM.

THE Cheltenham District Nursing Association seems to be carried on with exceptional energy and success. A short time ago an arrangement was made by the association with the Cheltenham Board of Guardians, under which the nurses attend to the requirements of the sick poor in the country parishes comprised within the Union, the staff being augmented for the purpose by two. After three months' trial there appears to be every reason to anticipate the happiest results. In that period, the nurses paid 1,457 visits to 70 cases in the country parishes, the patients being confined to persons in receipt of outdoor relief. Here is an object lesson in support of the contention that it is worth the while of Boards of Guardians to subscribe to district nursing associations. At Cheltenham the Guardians contribute £100 a year, and though this obviously does not cover the cost of the additional work, it is a material help; while the record of the work done supplies an argument on behalf of the appeal to those of the outside public whose names are not yet on the subscription list.

### IMPROVING THE TRAINING AT LIVERPOOL WORKHOUSE INFIRMARY.

The Liverpool Guardians, thanks mainly to the efforts of the late Mr. William Rathbone, have always been in the van of the movement for raising the standard of nursing the sick in workhouse infirmaries. We are glad to observe that they are resolutely maintaining their reputation. A special committee was recently appointed to consider the advisability of increasing the staff in the training school for nurses, in which there are now nearly a hundred probationers. This committee having reported that in the interests of the parish and of the probationers, it was desirable that the teaching and superintending staff should be strengthened, recommended the appointment of the present night superintendent as assistant day superintendent, with the promotion of an experienced staff nurse to the office of night superintendent. The Workhouse Committee of the board promptly adopted this recommendation, and acted upon it. Miss Thorburn, one of the lady guardians who has been very much to the fore in the matter, addressing the meeting at which the decision was arrived at, said she believed that the change would enable the probationers to be better trained, and Mr. Crosfield mentioned the interesting and suggestive fact that last year the Liverpool Guardians received £470 for the training of nurses.

### NURSING AS A VOCATION.

MISS MARION E. SMITH, superintendent of nurses at Philadelphia Hospital, read an interesting paper on "Nursing as a Vocation" at the 16th Annual Council of the Guild of St. Barnabas, in the course of which she insisted that a woman with a worldly heart and mind cannot be an ideal nurse. "The life of a nurse," continued Miss Smith, "is a hard one, especially that of a private nurse. She has many trials and temptations, long monotonous days and nights, the unreasonable relatives of the patient and the quarrelsome servants, a burden of anxiety, and very hard work besides. What has she to look forward to between cases? As a rule, a dreary boarding-house, often overcrowded, or two beds rented by four nurses. This is not uncommon in Philadelphia, at least. Under these conditions, if nursing is simply a means to an end, how can the enthusiasm and unfailing sympathy and patience last if the woman has a worldly heart and mind?" Miss Smith freely admits that skill and conscientiousness are the most important things in nursing, and that not even religious fervour can make up for incompetency; "but," she adds, "given an equal education and intelligence, the good woman—in other words, the Christian woman—makes the highest type of nurse, the woman who can be depended upon to be absolutely honest in words and purpose, unfaltering in her devotion to duty, dignified and gentle in conduct."

### MATERNITY NURSING IN RHODESIA.

MISS J. B. CROOKE sailed for South Africa on Saturday. She has gone out under the auspices of the South African Expansion Committee in order to undertake the duties of head maternity nurse at Salisbury, Rhodesia. Miss Crooke was trained at Chorlton Union Infirmary, West Didsbury, Manchester, and has since been ward sister at St. Leonard's Infirmary, Shoreditch, and charge nurse at the Fever Hospital, Tooting. Of course, she holds the L.O.S. certificate, and we believe that her appointment is regarded as one of considerable importance.

### The Nursing Outlook.

"From magnanimity, all fear above;
From nobler recompense, above applause;
Which owes to man's short outlook all its charm."

### PROGRESS AND THRIFT.

In our first number for 1903 we desire to wish all our readers, and especially those who are zealous workers in the nursing field, a happy and prosperous New Year. Christmas in the hospitals is a busy time of really arduous toil for matrons, sisters, and nurses especially. The work is a labour of love, and is now so general and so excellent in quality that in the hospitals probably more than anywhere else is the Spirit of Christ most manifest at this season of the year. We wish it were possible to lead all the prosperous, the selfish, the unthinking, and the idlers amongst us during some portion of Christmas Day into some ward of a great hospital. There they would realise, possibly for the first time, the joyousness of ministering to the happiness of their less fortunate fellows, and so learn to live rather than to exist. It is becoming customary with many people to spend a little time during Christmas week in one of the hospitals, and we hope all who follow this elevating custom may not forget to hand their contribution towards the expenses of Christmas-tide to the matron or the sister of the wards they visit. We hope that the choicest blessings of the new year may be poured upon all who have given of themselves this Christmas to cheer the sick and suffering in our hospitals and infirmaries.

We have decided to devote in future one page in each week's issue to the consideration of some question of special interest to the nursing world at the moment, or to one which deals especially with the higher branches of nursing economy and administration. There never was a period in the history of nursing when it was more desirable to crystallise sound opinion on the many subjects which affect the whole body of nurses. The present scandal in the greatest society of private nurses, The Nurses' Co-operation, is a matter which demands a speedy remedy. There are, of course, evils still to be redressed in certain institutions where the management is not up to modern standards, but on the whole there has been marked improvement all along the line. Considerable improvement has taken place in the mode of selecting women who desire to become probationers, and in the early stages of their training. The accommodation and food provided for nurses in hospitals have greatly improved. The plan of examination and the courses of lectures in the theory and practice of nursing have developed in recent years, but with the enormous increase in the number of women who become certificated nurses it is not a little remarkable to find that the difficulty of selection for the higher posts has tended to increase rather than to diminish. The cause of this is not far to seek. At present those trained nurses who are best educated and most suitable to take the higher posts have in this country no means whereby they can procure training in the theory and practice of much pertaining to the duties of a matron of a large hospital.

Again the Royal National Pension Fund for Nurses is one of the most gratifying successes of modern times. It has attracted to itself much that makes for the highest character and the highest success in a nurse's career, but that success has caused certain astute canvassers to endeavour to attempt to sell to the nurses various forms of insurance, backed by reckless promises in the way of bonuses, which experience proves cannot be fulfilled, and so to impose upon the members of one of the hardest callings which a woman can follow. The average commonsense of the British nurse has proved sufficient to render such attempts but little successful.

Having regard, however, to the fact that the actual insurance rates charged by the Pension Fund are 2 per cent. less than those of other insurance offices, and that the nurses who join the Pension Fund secure every advantage which co-operation can assure to them on the money they invest, we earnestly advise every nurse who is approached by an insurance agent, before entering into any contract, to communicate with the Editor of The Hospital, sending up the prospectus, the statements and promises received from the canvasser with whom they may be in communication. We will then make it our business to have the actual results tested by an actuary, so that every nurse may know exactly what she may expect before she commits herself to any contract of the kind. All respectable insurance companies admit that the advantages offered to nurses by the Pension Fund are on the whole better than any return which a nurse can obtain elsewhere on her savings. Any nurse who is a member of the Pension Fund may rest assured that by the exercise of moderate thrift she will be provided for for the rest of her life—an advantage which should inspire her with confidence to devote her whole energies to the noble profession to which she belongs. We hope that every nurse who is not at present a member of the Pension Fund will commence the new year by obtaining a prospectus and beginning to save something every month as a provision against the day of sickness and old age.

The Editor is always anxious to supply his readers with information upon any subject in which they may be interested. We shall therefore welcome suggestions of subjects which may be dealt with editorially on this page. We hope to have the co-operation of matrons, sisters, and nurses throughout the country, and indeed throughout the Empire, for the circulation of The Hospital is now so large, and the friendship begotten of many years' association which has grown up between the Editor and his readers is so real and good a thing in itself, that we are confident everybody, who has the best interests of the nursing profession at heart, will cordially co-operate to make this new departure in the Nursing Section of the paper a genuine success. We hope our readers will remember that nothing is too great or too small, providing it be a matter of interest to any section of our readers, to ensure for it the most careful attention at all times.

### Lectures to Nurses on Clinical Chemistry.

By CARSTAIRS DOUGLAS, M.D., B.Sc., F.R.S.Edin., Professor of Medical Jurisprudence and Hygiene, Anderson's College Medical School, and Dispensary Physician, Samaritan Hospital, Glasgow.

### LECTURE III.

DEPOSITS IN URINE (*Continued from page* 166).

3. *Mucus.*—This is a common deposit even in healthy urines. It has the appearance of a filmy semi-transparent cloud, occupying the lower fourth of the test-glass. It may be much increased in catarrh or inflammation of the bladder.

4. *Phosphates* very frequently form a distinct, even abundant, sediment in urine, and the nurse may note that the reaction in these cases is almost always neutral or alkaline. The deposit is white, and the phosphates present are generally those of lime or magnesia. Such a sediment is dissolved by an acid (*e.g.*, nitric or acetic), but is not cleared up by heat or by the addition of an alkali, like liquor potassæ. We often find phosphatic deposits in inflammation of the bladder.

5. *Blood* when abundant will form a distinct reddish-brown sediment, while the urine above is red or "smoky."

6. *Oxalate of lime* may also be mentioned. It is rarely abundant as a deposit, and is usually crystalline. The tiny crystals becoming entangled in the mucus cloud, give it a slightly glistening appearance, and this is sometimes termed the "powdered wig" deposit. It may be seen after a patient has had a hearty meal of stewed rhubarb.

7. The last deposit we need name is *pus.* It may be abundant, forming a heavy whitish-yellow collection in the test-glass. It somewhat resembles phosphates, but does not dissolve when an acid is added. We shall describe a chemical test for it later on. Pus is common in many forms of renal and bladder disease, especially cystitis, and tubercular disease of the kidneys.

### CHEMICAL TEST FOR PATHOLOGICAL SUBSTANCES IN THE URINE.

1. *Albumin.*—This is the most important of the foreign substances that commonly occur in urines in disease, and the one most frequently tested. In health the albumin of the blood (which is a kind of coasin of white of egg) does not find its way into the urine, but in disease of the kidneys it may, and therefore its presence, and the condition of "albuminuria" is a very important matter to determine. All the tests depend on the fact that this substance is coagulated (curdled as it were) by heat or by certain chemical reagents. So we may describe and consider three tests based on this.

(i.) Take one inch of urine in a test-tube, and heat gently in the spirit flame till nearly boiling. Note if any cloudiness whatever appears. If in doubt take a second test-tube and place another sample of urine in it. Do not boil, but hold it up for purposes of comparison. If still you are satisfied that no opacity is present, add to the heated urine a drop or two of acetic acid. It sometimes happens that the albumin is present in the form of alkali-albumin, and this is not coagulated by heat like ordinary albumin. If, however, a little acid be added so as to neutralise this alkali, we do get a precipitate of ordinary albumin, and it is for this reason that we use the acetic acid. In any case, however, some acid should be added after boiling, for it not infrequently happens that a cloud is produced on boiling a urine, which, unlike an albuminous coagulum, vanishes when an acid is added. Such a cloud is due to phosphates, which may in a neutral urine, be readily thrown down on heating. But phosphates, as we know, dissolve in acetic acid, while albumin does not; therefore we add the acid in all cases after boiling. If, after boiling and subsequent careful acidulation the urine remains clear, one is entitled to say no albumin is present.

(ii.) The cold nitric acid test (Hiller's). Nitric acid, like many other bodies, can precipitate albumin without heat. To do this test we place in a test-tube half an inch of pure, strong nitric acid and allow the urine to trickle gently down upon it, preferably by means of a long glass tube, called a pipette. In the presence of albumin a white or dull opaque ring of coagulated albumin forms where the two fluids join. This test is delicate and easily performed, but is not free from fallacies. If the urine is very concentrated the urea (or chief solid in it) may, on the addition of the acid, separate out as a flat cake of crystals of nitrate of urea, resembling a coagulum of albumin. It dissolves, however, with heat, and if the test be peformed again with the urine diluted with an equal volume of water, the fallacy will not occur. Again, when nitric acid is added to a urine of a patient who has been taking certain drugs, such as copaiba, cubebs, styrax, balsam of Peru, etc., a coagulum may appear, due to the effect of the acid on the drugs excreted in the urine. Such drug opacities are cleared up by adding a little spirit.

(iii.) A third and final test for albumin is to place a little urine in a test-tube and let a little of a saturated solution of *picric acid* trickle down on it, when, if albumin be present, a yellow coagulum will form where the two liquids touch. This test is very delicate.

*Tests for Sugar in Urine.*—Hardly less important than albumin is sugar, as a foreign element in urine. Even in health, it is true, minute traces of sugar are thrown off by the kidneys, but in amount too small to be detected by the ordinary tests. The sugar that is found in disease is grape sugar or glucose, not cane sugar (saccarhose), and it is *the* important substance and the diagnostic indication in diabetes mellitus. One must not imagine that every patient whose urine contains sugar is a sufferer from diabetes, for it may occur in gout, in dyspepsia, and under certain other circumstances. But when it is considerable in quantity, persists in spite of dietetic treatment, and is accompanied by great thirst and voracious appetite, we may feel assured we are dealing with the genuine disease.

*Tests:* (1.) That employed perhaps most frequently is Fehling's, and the reagent, named Fehling's solution, is a solution of tartrate of copper containing a large quantity of alkali (caustic soda). It is a liquid of a beautiful deep blue, which when boiled with a sugary urine throws down a distinct reddish-yellow opaque precipitate of what is called cuprous oxide. Fehling's solution does not keep well long at a time, and may, if old, develop something in it which on boiling may produce a precipitate much as sugar does. So, in performing the test, a nurse should always take a little of the solution (say half an inch in a test-tube) by itself first and boil it thoroughly. If it remain clear and blue, the solution is good; if it become opaque and greenish or yellow, it is not reliable. Having satisfied herself that the liquid is right, the nurse should now add a little of the suspected urine and boil again, when in the presence of even small amounts of sugar the typical yellowish-red precipitate appears, or, as it is termed by chemists, the Fehling's solution is "reduced" by the sugar. It should be added that Fehling's solution may be made up in separate portions—one bottle containing sulphate of copper, the other an alkaline solution of a tartrate. These solutions keep better than Fehling's reagent, and when required for actual use need simply to be mixed in equal volumes.

### Christmas in the Hospitals.

BY OUR OWN COMMISSIONERS.

IN our Christmas number we were able to show, both by letter-press and by illustrations, that the Christmas festival is now observed in the leading workhouse infirmaries with an enthusiasm and a whole-heartedness which leave nothing to be desired. The accounts that follow of the entertainments in the London and other hospitals, justify the assertion that there is no slackening in the pioneer institutions of the sustained and successful efforts on the part of the medical and nursing staffs to afford gratification to the patients under their charge.

Novelties in the form of decoration or entertainment become increasingly difficult, because with each succeeding year the resources of all concerned have been drawn upon so largely that originality must almost be exhausted. At one of the most popular hospitals a new feature was introduced into the decoration of a children's ward, and the little inmates were delighted by the erection of two brightly-coloured maypoles which were also utilised for the support of long festoons of evergreens. At another, "The New Year 1903," a nurse in white accompanied Mother and Father Christmas; and at a third, a nurse in Chinese dress, speaking Chinese, arrived apparently unexpectedly, and distributed presents. The fact is that originality has ceased to be essential, owing to the high pitch of excellence which has been attained, whether in the transformation of the wards, the selections of the songs, the arrangement of the menu, or the choice of the gifts. It must be recollected that though the programme may not change much as the festival recurs, it is almost always fresh to those for whose benefit it is planned. Although the visitors recognise familiar features, there is obviously one overwhelming argument in favour of the repetition of an entertainment. If it has been greatly appreciated by a set of patients one year, there is every reason to believe that it will equally gratify another set the next year. Practice, moreover, cannot fail to tend towards perfection, and slight alterations, judiciously made, are better in such circumstances than a brand new scheme imperfectly thought out and adopted merely for the sake of novelty. There are certain attractions which, indeed, can never be dispensed with. It is not every hospital which has a kindly doctor who assumes the character of Santa Claus, or a clever band of students who convert themselves for the time being into nigger minstrels, but the Christmas tree for the children and the singing of carols by the nurses are items which could not be eliminated without spoiling the festivities. One wonders sometimes whether it is sufficiently impressed upon the patients of the part which they should play on the occasion, how much depends upon the spirit of cheerfulness, the determination to be pleased, the desire to give no unnecessary trouble at an exceptionally busy time, which they exhibit. It is, in a word, their obligation to supplement the efforts of willing workers who spend precious intervals of leisure for many weeks beforehand in thinking out the scheme, and putting their ideas into execution. But, at any rate, the public have the satisfaction of knowing that the victims of accident or disease who have to spend their Christmas in the wards, enjoy substantial compensation—so substantial that men and women, as well as children, often express their thankfulness at their enforced detention.

### THE LONDON HOSPITAL.

Christmas Day began early for the patients in the London Hospital, cards and letters being distributed by the night sisters before they went off duty. Then came carols by the sisters, nurses, and probationers, good Christmas cheer at dinner-time, when some of the wards were fortunate enough to have turkeys, sent by friends of the hospital; and great delight was given in many cases when "my own doctor" brought round the pudding. From 3.30 to a few minutes after seven an elaborate programme of entertainments was gone through, thirteen wards being visited by as many "troupes," including "The Pink 'Uns," Herr Koph Schmartz's "Band," "The Wise Men of the East," "The Wot Nots," "Miss Julia Mackay's Troupe," "Miss Mukle's Instrumental Troupe of Ladies," "Oxford House," and others. Currie Ward was the scene of the first performance of the "Wot Nots," who consisted of half-a-dozen sailors and their lady-loves, with voices of a tell-tale bass. Songs, dances and banjo duets were in the programme, and the singing of a topical poem, wherein occurred "I'm glad I reside on the surgical side," and the statement that the nurses were harder to deal with even than the doctors caused a great deal of laughter. Gloucester Ward was meanwhile enjoying Herr Koph Schmartz's band, a mixed medley of ladies, a policeman, a monk, a judge, a Chinaman named "Ping-Pong," a Scotsman in Highland dress, and a coster supposed to be fetched in from Whitechapel Road to accompany the strange instruments on the piano. During the performance a telegram "from the Kaiser," regretting his inability to be present, was read by Herr Koph, whose band was said to have performed before all the crowned heads of Europe, including the chairman of the London Hospital. The patients, drawn up in rows in their beds, showed their appreciation of the performance in hearty clappings, and the troupe departed to the strains of "God Save the King" in as many different keys as there were instruments. By four o'clock the troupes were hard at work; "The Farce" was in the New Block, "Miss Mukle's Troupe" was in Currie, "The Wot Nots" in Talbot, an Impromptu was going on in Charlotte, in Victor Ward Mr. Seymore Dicker's Troupe, consisting of Miss Nellie James, Miss Lilian Gosling, Dr. Graham Grant and Mr. Seymore Dicker, was entertaining the patients; Miss Julia Mackay and her assistants were in Sophia Ward; Mr. Dodd's Party, consisting of Mrs. Keith and Mr. Dodds, was in Rachel; Mellish had a Conjuror, Cotton and Mary had the "Pink 'Uns" and "The Savoy;" "The Wise Men of the East" had superseded Herr Koph Schmartz's Band in Gloucester, and the lively conductor was repeating his jokes in George Ward, while Rowsell was being highly amused by a good Gramophone and "Oxford House." By means of careful organisation and really hard work on the part of the troupes, every ward had a succession of half a dozen entertainments, each lasting about half an hour. Many friends of doctors and nurses visited the hospital during the afternoon. The wards were prettily decorated with evergreen and flowers, and the fumes of the unwonted pipe in the men's wards added to the holiday feeling that pervaded the whole of the vast building.

### GUY'S HOSPITAL.

Guy's has a distinct style of its own with regard to ward decorations, and each of the 24 was a picture in itself. By means of electric wire coils, which are kept from year to year for the purpose, delicate trailing ivy, with coloured electric lamps at intervals, was suspended across the ceilings and fastened to cot-heads; Astley-Cooper and Cornelius were perhaps the prettiest, with their traceried curtains of evergreen inside the doorway; but all, even Patience, with its gable roof and narrow limits, were graceful and Christmas-

### CHRISTMAS IN THE HOSPITALS—*Continued.*

like for a whole week. Chinese lanterns and fairy lamps were used in addition to the electric light in some wards, and in Martha there were some simple but most effective butterfly shades make of crinkly paper. The chapel was equally well decorated, and services were held at 6, 7, and 8.45 (Holy Communion), and 11 (Matins) on Christmas Day. Every patient received a present, and the children a stockingful of toys, while all the full-diet patients had roast beef and vegetables for dinner, followed by plum-pudding and dessert. Smoking was allowed on Christmas and Boxing Day, and on the occasion of ward concerts. The students went through their minstrel troupe performances during the afternoon in the wards, and the annual out-patients' tea was given to about 600 poor children from the crowded district which surrounds the hospital. This was a successful, if somewhat noisy party, and great delight was afforded by a series of cinematograph pictures and Punch and Judy, which, with a performance of the minstrel troupe, formed the entertainment. There were two enormous Christmas trees, loaded with toys, dolls, fruit and sweets, which were distributed among the happy crowd, and as the guests departed, each was given a parcel containing a loaf of bread, tea, sweets, apples, oranges, crackers and a useful article of clothing. The cost of the "Out-patients' Tea" is covered by a voluntary fund, to which those who work in the hospital and their friends contribute. During Christmas week a concert was given in each of the surgical wards, and the songs, violin solos and glees, interspersed with ventriloquism, sleight of hand, musical sketches, and "chapeaugraphy" (a clever piece of change of personality by means of a hat) were a source of much pleasure to the patients, and fully justified the notices that caught the eye of the visitor—"The way to Luke is shorter and the concert is much better"; "We can seat hundreds, come in thousands," etc. On the evening of Boxing Day and on Saturday the resident staff gave an entertainment to the nursing staff and friends, of which "A Comedy of Courtship" (an episode adapted from "The Philosopher in the Apple Orchard," by Anthony Hope), "The Ghost of Jerry Bundler," by W. W. Jacobs and Charles Rock, and "The Area Belle" were the chief features. In place of giving concerts in the medical wards, a united medical wards concert was given on Tuesday (after Christmas) to which all the medical patients who were well enough were invited. This was held in the out-patients' hall.

The Christmas tree in Martha ward, on Saturday afternoon, was as great a success as ever. The ward was exceedingly pretty, and the decorations already described culminated in a magnificent tree, lighted by electricity and loaded up to the ceiling with toys. Tea, dispensed by the students and nurses, formed an interlude to Punch and Judy, which, as it is rather long, was better for being given in two parts, especially as some of the babies were very small indeed. Then came some glees and songs, and finally the event of the afternoon—the arrival of Father Christmas, who was greeted with rapturous applause, and who went to work at once in a thoroughly businesslike way. The first gift, which he hastened to present with great ceremony, was a doll for the Matron, and then the children's gifts came in rapid succession, until not a toy remained on the tree, and the sound of drums and trumpets was heard in the land. All the children had a good time, but perhaps Hilda, aged about two, with a bandaged foot, was the greatest pet of all, and every nurse had a turn in carrying her about the ward.

"Do you think all this excitement is bad for the patients?" someone asked the Matron. "It does not seem to hurt them," she replied, "and they sleep the better for it."

### St. Bartholomew's Hospital.

There is a strong feeling among the authorities of St. Bartholomew's Hospital that a great deal of festivity is out of place in an institution devoted to the sick and dying, and with this view the matron thoroughly concurs. Accordingly everything was done this year to keep the patients as quiet as possible, and no entertainments were given except those in the wards. And here, judging by a visit to two of the women's wards about 5 o'clock on Christmas Day, there was plenty to amuse, and no possibility of dulness, while strains of "Noel, Noel," floated pleasantly from the neighbourhood of "Mark" ward into the courtyard, and many lights shone from the sides of the quadrangle. The women in Stanley ward were being amused by a ventriloquist, and a tiny patient of about three was standing in front of Tommy and the old lady, evidently wondering what they were made of. Of course the old lady recited "Twinkle, twinkle," quite incorrectly, with frequent allusions to that shilling that she never seems to receive, and of course Tommy made a great many irrelevant remarks, and was rebuked in the usual way. But both puppets uttered quite the right sentiment when they said they "did like Bartholomew's;" and no doubt the remark was echoed throughout the ward. When they retired to their box they were followed by a conjuring entertainment. Moreover, there was a Christmas tree, coloured paper chains were festooned across the ceiling, and Sister Stanley may be congratulated on the very bright and pretty appearance of her ward. In Lawrence ward a venerable Santa Claus was meanwhile going from bed to bed distributing the gifts cut from an enormous tree at the further end of the ward, over which Sister Lawrence was presiding with a group of nurses round her. A novel personage, the "Duke of Wellington," had already visited one or two of the men's wards, and, in some form or other, presents had been received by all the patients throughout the hospital.

### King's College.

Monday, the 29th, was the great day at King's College Hospital, and certainly the students and nurses must have worked very hard, for they were engaged in entertaining the patients the whole afternoon, and only finished their rounds at about half-past seven o'clock. The first thing that attracted the notice of the visitor was a curious object in the entrance hall, which, upon examination, proved to be a statue turned into a Santa Claus for the time being. Sounds of merriment came from upstairs, and strange forms passed from ward to ward, pausing for a brief rest before proceeding to the next performance. There were two sets of performers, "The School" and "The Band," and both were highly entertaining. The particularly naughty children, of a preposterous size and infantile appearance, whose manly voices showed through the thin disguise, were presided over by a voluble schoolmaster of terrible energy, who was as easily hoodwinked as a child. The fat boy, who industriously ate carrots and apples, the "baby," who was poisoned by the contents of a doubtful-looking bottle, and had to be taken off post haste to King's College Hospital, were striking figures, but they were eclipsed by the evilly-disposed "Franky Fergusson," who arrived late with a note from his mother and a medical certificate from "the 'orspital," to the effect that another boy had walked on his "nasal organ," which was appropriately bandaged. This engaging youth was armed with a bottle (the same that poisoned the baby) dangled on a string, and a kipper to which he seemed specially attached. The minute plum-pudding, intended as a surprise to the class, and which was garnished with a very large sprig of holly, was carried off by the fat boy while the master sketched it on the blackboard, and the whole school slunk out in its wake, to the sound of hearty applause, and was followed by the master, dolefully shaking his head. Then followed a selection of songs by Sister Clare, Nurses Michist, Clapperton, and Smith, and Messrs. Mansfield, Anderson, Napper, Young, and Clay, and a violin solo from Sister Ethel. The band, meanwhile, was performing hard by, clothed in what had every appearance of being patients' scarlet jackets, and white leggings bound with red tape, their disguised faces being surmounted by white turbans trimmed with two long pheasant's tail feathers or scarlet wings. Their instruments, though sounding much alike, were of various strange shapes, and when the music refused to come were medically examined, and found to contain many yards of coloured streamers. The agile dances of the conductor and "Captain Kettle," and the songs, recitations, and "pieces" were warmly applauded, and evidently gave great pleasure. The wards were festooned with evergreens; art muslin, flowers, and coloured lights on the mantelpieces and window-sills, and the red sprays and green tracery of one or two wards all imparted a holiday aspect to the hospital. In the children's ward two cleverly coloured Maypoles were turned to account for decorative purposes. The male patients, in scarlet bed-jackets, pipe in hand, looked the picture of comfort, as they lay and watched the truly laudable and successful efforts of the students to provide them with amusement. The nurses had their Christmas dinner on Saturday, and on Tuesday there was a Christmas tree in the children's ward.

### CHARING CROSS HOSPITAL.

Thanks to the forethought of Miss Smith, who is taking temporary charge of Charing Cross Hospital, the wards were as pretty and bright as ever; roses and chrysanthemums, fairy lamps, and many kinds of cakes and sweets, decorated the ward tables, while trailing evergreens, Chinese lanterns and flags were festooned across the ceilings, and sprays of greenery beautified the walls, harmonising with the scarlet dressing-gowns of the patients, by whose beds friends sat and chatted during the afternoon of Christmas Day. The celebrations began on Christmas Eve, when the nurses sang carols in the corridors, and there were carols again from the choir boys of St. Martin's Church during the afternoon of Christmas Day. In the morning each patient received a gift, and at tea-time a party of ladies and gentlemen gave songs and music, and a "coon song," of which the chorus was remembered, at any rate by the staff, from last Christmas, gave great delight. Then there was a clever little gipsy song from a pretty girl in red and black with a tambourine, which was composed by the performer of the coon song. The entertaining in the wards was of an impromptu character, and from the faces of the patients sitting up in bed it was evident that this form of amusement gave great pleasure. *Truth's* doll, larger than ever, had a place of honour among the children, and a family party, of which the mother was the patient, were pulling an enormous cracker which startled the ward with its miniature explosion. Upstairs, the father of one of the patients was the fortunate possessor of a gramophone, and the performances of this instrument were very much enjoyed. Later on there will be three entertainments in the board-room, to which all the convalescents will go down.

### WESTMINSTER HOSPITAL.

Lucky-tubs are the speciality at Westminster Hospital, and the most original one this year was evolved by Percy ward. It was in the form of a well, about two feet of the brickwork being very cleverly imitated, and planted with real ivy, on which frogs hopped and spiders hung. Above this was the wooden pent-house roof, also festooned with ivy in a very naturalistic way, and on the roof were the words, "Ding, dong bell, Pussy in the well." Pussy was really in the well, for when the handle was turned and the rope wound up, there she was, clinging to the bucket, which, of course, contained somebody's present. Altogether, it was a very clever bit of work. "I wonder," someone remarked to Sister Percy, "whose idea it was?" But she only echoed, "I wonder," and was not to be drawn. Arden ward had another very pretty tub covered with dainty crinkled paper flowers, and it was here, too, that the "prettiest table in the hospital" was to be seen. Scarlet carnations, pointsetzia, and bavadia, relieved by delicate asparagus fern, were arranged on pale green art muslin tied with small pompons, with a very pretty effect. The other wards each had their special scheme of decoration, with evergreens and coloured lamp shades, and there was a tree for the children as usual. At half-past ten on Christmas morning there was a visit from Santa Claus, whose progress through the wards was accompanied by the lively strains of a street piano—a novelty for which even the sisters were unprepared. Then came the Christmas dinner, and after a short interval the fun began again with ward teas and an entertainment in the Board Room at four o'clock. This consisted of a farce acted by two grandsons of Charles Dickens, followed by an excellent musical programme. Up to seven o'clock the festivities were still going on in the incurable ward, where the house surgeons were giving an impromptu performance of such old favourites as "The bonnie banks of Loch Lomond" and "Off to Philadelphia in the morning." The sisters had received an invitation to the house of Mr. Dickens (son of the novelist) to hear his reading of the famous "Christmas Carol," after the festivities were over, and Sister Arden was heard to remark, "Christmas at Westminster is always nice, but it has never been nicer than to-day."

### ST. MARY'S, PADDINGTON.

On Christmas morning every patient received a present. The children had toys and sweets, the adults some useful article of clothing; the male patients were given the wherewithal to smoke. Carols were sung in the hall on Christmas Eve and in the hospital chapel on Christmas Day by the choir. The wards were simply but tastefully decorated. Pink was the favourite colour in De Hirsch, the children's medical ward; scarlet in Crawshay, the children's surgical ward. All patients sufficiently well had either turkey, chicken, or pheasant for dinner, followed by plum pudding, fruit, etc. A piano was provided for nearly every ward, and the friends of patients visited them from 3 to 4 P.M. At 4 o'clock the festivities commenced with a liberal tea, followed by songs and recitations. All the patients enjoyed themselves thoroughly. There was a Christmas Tree on Tuesday for the children in the board-room.

### CHELSEA HOSPITAL FOR WOMEN.

Owing to the kindness of Dr. and Mrs. Fenton every person in the Chelsea Hospital for Women—nurses, patients, and servants—received a really good and useful gift on Christmas Day. Each of the corridors was gay with festoons of evergreen, interspersed with Chinese lanterns and coloured shades on the electric lights, while rows of fairy lamps stood on the floor. These were presented by Messrs. Clarke, the well-known candle manufacturers, and when lighted up had a very brilliant effect. On one of the floors the nurses had cut out and fastened on the wall a large white swan of cotton wool, with real white turkey feathers, and below was a miniature pond with more swans. In the wards the available funds had been partly spent in white flower vases of various designs; the electric lamps were covered with many coloured shades, and a little evergreen beautified the walls. Nurse Kingdon, in charge of a floor, was most ingenious in thinking out and preparing in her off-duty time a series of nursery rhymes. In one ward was a very succcessful "Mad Tea-party" on a table all to itself. The Mad Hatter, for whom a special doll had been made, was quite correctly habited in black and white check, and his hat bore the legend: "In this style 10s. 6d." Alice was a very pretty little doll, and the March Hare had quite properly bound his ears with hay and gauze, while a minute dormouse slumbered at his side. Another ward had the "Babes in the Wood," and then there was a scene in fairyland, from "The Midsummer Night's Dream," with Bottom in his ass's head, and Titania crowning him with garlands. On another ward table was a Japanese scene. On the chimney-piece was Dick Whittington journeying to "London, IV. miles," accompanied by his cat. Each scene had its appropriate setting of sprigs of evergreen and flowers, in one case cowslips obtained from crackers. Downstairs in the Board Room flags were suspended above the table, and evergreens were on the walls; and here, after the labours of the day, the nurses dined with the matron. The Ladies' Committee sent numberless good things to eat. There were turkeys also in the wards, and to a visitor who said she wished she had brought some of her turkey and plum puddings, one of the patients answered, "Oh, you needn't trouble, we've had turkeys here!"

A beautiful hammered silver fruit dish was presented to Miss Heather-Bigg by the nurses on Christmas Day, in view of the matron leaving the hospital early in the new year. On the 30th a patients' tea was given by Mrs. Frank Stone, when all the waiting was done by the ladies of the party, the nurses being made to take a rest. Some of the members of Lady Edward Moss's Band kindly gave a selection of music, and the nurses sang glees. On January 3rd the Ladies' Committee will give a tea, when each nurse will have the privilege of inviting a friend.

### Mount Vernon Hospital for Consumption.

The vein of originality at the Mount Vernon Hospital, which last year produced a "Mother Christmas," has this year added one more to the group of personalities. "The New Year, 1903," in the form of a pretty nurse in white, accompanied the aged couple through the wards and open-air tent in the grounds, and helped to distribute the gifts on Christmas Day. The wards and corridors, open to the winds of heaven, as they always are, were gay with coloured "crêpe" paper lamp-shades and evergreens strung on red braid, the latter being confined to the corridors and hall. For a great part of Christmas Eve nurses and patients had been at work beautifying the hospital, and the effect was very bright and pleasing. As one entered "A Happy Christmas" confronted one in the hall, and this was the key-note of the whole. All who were well enough dined in the large dining-room, to the accompaniment of music, and the table, well spread with good Christmas cheer, was decorated with dainty baskets of fruit for dessert. Later on in the day there was more music, and on Saturday, beginning at 6 30, there was a concert and conjuring entertainment followed by a festive supper. On Monday came the turn of the nurses, when an entertainment, at 8 o'clock was followed by a dance. The servants had their entertainment on Wednesday evening. The cost of presents, etc, for the patients is defrayed out of a voluntary fund, to which the committee and other friends of the institution at Hampstead contribute.

(*To be concluded next week.*)

### Our Christmas Distribution.

The following letters have been received by us from the recipients of parcels of garments which we were able, through the thoughtfulness and generosity of our readers, to distribute:—

The Matron of Guy's Hospital, S.E., writes:—"I thank you very much indeed for the gift of garments you so kindly sent to the hospital for the patients. They will be much appreciated. Will you please thank your readers for their kind thought."

The Matron of King's College Hospital writes:—"We wish to express our very warm and grateful thanks, for the parcel of nice Christmas gifts received to-day from the Editors and readers of The Hospital for the patients of this hospital. It is indeed a great kindness, and we wish our generous friends could witness the pleasure that is caused to the poor sick when they find what a substantial greeting is in store for them on Christmas morning."

The Secretary of St. George's Hospital, Hyde Park Corner, writes:—"I beg to acknowledge with many thanks the very kind present of clothing for the use of the patients from the editors and readers of The Hospital."

The Matron of St. Mary's Hospital, W., writes:—"Allow me to thank you most warmly for the useful gifts you have so kindly sent for our poor patients. We are most grateful to you for them and they will be much appreciated."

The Matron of the Metropolitan Hospital, Kingsland Road, N.E., writes:—"We acknowledge with very grateful thanks the articles you so kindly sent to us; they will be most useful, and we appreciate your kindness in thinking about us, very highly, for we need warm clothing for our patients very much, and it is such a comfort to be able to give them a warm garment to wear when they leave the hospital."

The Matron of Victoria Park Hospital, E., writes:—"I thank you very much indeed for the kind gift of warm clothing for the patients. They were doubly welcome as so very few things have been sent to the hospital this year."

The Secretary of the East London Hospital for Children and Dispensary for Women, Shadwell, E., writes:—"I beg to acknowledge the receipt of yours of yesterday, and on behalf of the Board of Management to thank you very sincerely for your welcome present of clothing which is much appreciated."

The Sister-matron of Paddington Green Children's Hospital writes:—"On behalf of the committee I beg to return you their best thanks for your kind gift of sixteen articles of clothing to the hospital."

The Matron of the Belgrave Hospital for Children, London, writes:—"I acknowledge with many thanks the receipt of twenty-two garments for the use of the patients when our new hospital opens."

The Lady Superintendent of the East End Mothers' Home, 394 and 396 Commercial Road, E., writes:—"Many thanks, indeed, for the most useful things you have sent for the patients in this home from The Hospital Christmas Distribution. The women will be delighted to receive them, and I am sure, if the readers and workers could see some of the miserable homes and children to whom their gifts will go, they would be more than repaid for their work."

The Matron of the British Lying-in Hospital, Endel Street, W.C., writes:—"I beg to acknowledge with best thanks a parcel of clothing for mothers and babies containing twenty-eight articles."

### The Civil Hospitals and the Army Nursing Service Reserve.

BY A RESERVE SISTER.

THE position of the members of the Army Nursing Service Reserve is a very serious one, and is giving them much anxiety, as the service is practically boycotted by the civil hospitals. This boycott is not of recent growth, but has existed since the first members were admitted in 1897. In that year a copy of the rules and regulations of the service was posted on the notice board in my training school, and the matron told the nurses who consulted her about joining the service that every nurse was free to do exactly as she wished in the matter, but that she should not advise any one to become a member, as membership of the A.N.S.R. would prove a bar to hospital appointments. The matron was perfectly right, and my own experience, and that of other Reserve sisters, has been that matrons of civil hospitals have either refused altogether to appoint a member of the A.N.S.R to a vacant post or have made it a condition that she resigns her membership on appointment. There are three points of view from which this condition of affairs must be regarded:—1. The effect on the service itself. 2. The reason of the matron's attitude. 3. Its effect on the members of the A.N.S.R.

### THE EFFECT ON THE SERVICE ITSELF.

It needs no great foresight to see that if the present attitude of the matrons of civil hospitals towards the service continues the A.N.S.R. must either cease to exist altogether or consist entirely of private nurses. It is impossible to maintain in time of peace a sufficient nursing staff to supply the needs of the Army in time of war, and so all (hospital matrons included) agree that the existence of a reserve of thoroughly trained nurses, to be drawn upon in time of emergency, is a national necessity. Great wars are happily rare, and one has to go back to the Indian Mutiny or the Crimean War to find a parallel to the strain cast on the whole resources of the nation during the past three years. It is only in a great war that the services of the Army Nursing Service Reserve are likely to be required, but when the need arises the nursing profession should be ready to meet it with a supply of the best nurses and most capable women it can produce—able to rise to an emergency and to nurse and control a large number of patients and orderlies under the very difficult, and to many untried, conditions of active service. It is in the wards of a hospital alone that such women can be found; yet the heads of the nursing profession are, whether consciously or not, making it impossible for the Army Nursing Service Reserve to obtain them. A private nurse is necessarily out of touch with hospital work; the nursing of one patient at a time is a very different matter to the management of a ward. Many women take up private work for the very reason that, though excellent nurses in every other respect, they do not possess the administrative qualities required of a ward sister. Civil hospitals recognise this fact by declining to appoint private nurses to vacant posts on their staffs. It is not a question of good nursing, but of fitness for a special kind of work—the work required of a sister of the A.N.S.R. when called up for duty.

### THE MATRON'S ATTITUDE.

The attitude of the matrons of civil hospitals towards the Army Nursing Service Reserve arises, without doubt, from their wish to avoid the inconvenience which would arise were many of their staff called away from their posts to serve their country at the same time. This difficulty has to be met by every employer whose employés are at their country's service in case of need, whether as reservists, volunteers, or even as remounts! But the matron's position is rather that of a trustee of a hospital than that of a personal employer of labour, and no doubt she feels obliged to subordinate her patriotic sentiments to the needs of her hospital. On the other hand, ought she not in her position of trustee, to consult the committee of the hospital before deciding whether such an extreme measure as the boycott of the A.N.S.R. is necessary in its interests? It is a serious matter, affecting a large number of the nursing profession as well as crippling the service. The difficulty of the matron is more apparent than real; for it would be a very simple matter to arrange that a certain percentage only of her staff could be allowed to be members of the A.N.S.R., and it would be perhaps only once in ten years at the very least that they would be likely to be called up for duty, a great war being a rare event. Perhaps if necessary an arrangement might be come to with the War Office either to pay the hospital the gratuity (apart from "batta" money) given to the sister at the termination of her period of duty in return for her post being kept for her, or to abolish the gratuity in the case of hospital sisters and pay the hospital a retaining fee of so much per annum for each member of the A.N.S.R. on its staff. The advantage to the War Office of this arrangement is obvious, as a thoroughly efficient reserve would be ensured. Also the members of the A.N.S.R. would feel the credit and honour of their service were in safe hands, as the matrons would practically have the choosing of it, and everyone agrees that the matrons, and not the medical men, are the best judges of the characters and capabilities of their nurses and their suitability for the service.

### THE EFFECT ON THE MEMBERS OF THE A.N.S.R.

The serious effect of the matron's attitude to the A.N.S.R. on its members' prospects in their profession can hardly be exaggerated. They must either resign their membership or give up all hope of hospital work, which is the only employment fitting them for their duties in time of war. Those sisters, whose posts at the hospitals were kept open for them, were hardly any of them Reserve sisters proper called up for duty, but merely joined the A.N.S.R. for their period of service, being required by the War Office to do so. The majority of the A.N.S R. on their return home from South Africa find that the doors of the civil hospitals are closed to them, and that there are practically no vacancies in the Q.A.I.M.N.S. for sisters of the A.N.S.R., as the supply of Army nursing sisters, according to your correspondent's accounts of the interviews with the matron-in-chief, already exceeds the number of posts in the new service. They are also uncertain whether, if again called up for duty, they will be given the temporary post of sister or be reduced to the position of staff nurse under a sister, in spite of having had some three years' experience on active service in the higher capacity in the old Army Nursing Service before the present innovation of staff nurses. One unpleasant subject cannot be entirely ignored in writing of the A.N.S.R. Amongst the 900 nurses engaged by the War Office in a time of emergency there were a few who brought great discredit on their profession and the service to which they belonged by the frivolity of their behaviour. The number of these cases was small, but was artificially magnified by the more than foolish action of the authorities in transferring these "undesirables" from hospital to hospital as complaints were made, instead of at once sending them back to England when they had proved by their conduct their entire unsuitability for the work of nursing sick and wounded soldiers in time of war. None but the most narrowminded and prejudiced person would condemn a whole service for the misconduct of a few of its members, and yet it is reported that the matron of an important London hospital has said that she would never engage a Reserve sister, no matter who or what she might be, and she should advise all matrons to follow her example.

### Everybody's Opinion.

**[Correspondence on all subjects is invited, but we cannot in any way be responsible for the opinions expressed by our correspondents. No communication can be entertained if the name and address of the correspondent are not given as a guarantee of good faith, but not necessarily for publication. All correspondents should write on one side of the paper only.]**

### "SHAM CO-OPERATION AT GLASGOW."

"MR. JOHN STEWART BANNATYNE, 138 Cambridge Drive, Kelvinside N., Glasgow," writes: I perused with much interest your note under the above heading. Although at the annual meeting it was understood that a print of the proposed constitution was to be sent to each of the nurses for approval that has not yet been done. The nurses who voted for the amendment, which was simply that the proposed constitution should not be adopted until the nurses had had a fair and reasonable opportunity of perusing and considering it, have since apologised to the executive for doing so. The executive, who have a weakness for apologies, should disclose to the public the terms of the apology, and whether it was made voluntarily.

### CHELTENHAM GENERAL HOSPITAL.

"CHAMPION" writes: I beg you will pardon me for again troubling you with correspondence on the above subject, but I feel that the letter in a recent issue, by the Hon. Sec. of the Cheltenham General Hospital, calls for some explanation. I am not aware that the message sent to the dismissed nurses, that if an apology was not forthcoming they must be prepared to forfeit their certificates, emanated from the Committee. The message was sent to them from the matron, by one of the senior nurses.

### CHRISTMAS DAY AT AN INDIAN MILITARY HOSPITAL.

"A SISTER" writes: We always try to make Christmas as happy a time as possible at the Secunderabad Military Hospital. Last year we gave all the patients a big tea. I was on duty from seven till twelve in the morning, and employed myself chiefly in getting the wards and verandahs ready for the festivities. The ward boys do not take as kindly to the menial part of the work as the good old much-abused pros. in a London hospital. We have a lovely long verandah all open, built on light pillars. By the rails we grouped pots of plants, chiefly lilies and ferns; in the middle we put a long table, tastefully decorated with vases of beautiful flowers (sent by the Colonel); in between were small dishes of sweets, crystallised fruits, oranges, and plates of fancy cakes, bread and butter, dishes of jam, etc., and for once our Tommies had their tea out of white and gold china cups and saucers. Those who were up sat round the table, but those in bed were not done out of the fun, for we brought them out beds and all, except two very bad cases. While they were having their tea and pulling crackers—which muscular exercise Tommie enjoys as much as any child, saying "the crackers are the best part of the show"—the band played selections from popular airs. Later, the General and several officers and their wives came by special invitation, and the ladies pulled crackers with the patients, and did their utmost to help to cheer them up. Then cigars were handed round, and those well enough to smoke did so, and others sang, accompanied by the band. All too soon the happy evening came to a close. "God Save the King" was shouted lustily, and one and all went to sleep happy and satisfied, saying "they never had spent a better Christmas."

### "The Hospital" Convalescent Fund.

THE Honorary Secretary acknowledges with best thanks the receipt of 2s. 6d. from Nurse A. H., who kindly promises to give the same amount each year if she can. The Honorary Secretary thanks Nurse A. H. for her nice letter, and is glad to hear that she is almost well again and very happy in her present post.

### Appointments.

CARDIFF WORKHOUSE HOSPITAL.—Miss Grace Wareham has been appointed charge nurse. She was trained at Holborn Union Infirmary, and has been assistant nurse at the Eastern Fever Hospital, Homerton, and charge nurse at Long Reach Hospital, Dartford.

ISOLATION HOSPITAL, CHESTER.—Miss Edith Priestley has been appointed sister of the scarlet fever blocks. She was trained at the Evelina Hospital, Southwark, London, and at the General Infirmary, Chester. She has since been doing private nursing.

MACCLESFIELD FEVER HOSPITAL.—Miss Louie Chapman has been appointed matron. She was trained at Little Bromwich, and the City Hospital, Birmingham, and for the last five years she has been charge nurse at the Isolation Hospital, Crewe.

PARKFIELD NURSING HOME.—Miss Jessie H. Grimshaw has been appointed lady superintendent. She was trained at Brownlow Hill Infirmary, Liverpool, and has since held the posts of home sister and superintendent nurse at Prescot Infirmary, sister at Watford Isolation Hospital, and home sister at the Children's Convalescent Home, West Kirby.

PORLOCH DISTRICT NURSING ASSOCIATION.—Miss Alice Anderson has been appointed district nurse of Porloch, Porloch Weir, and Bossington, in Somersetshire. She was trained at Paddington Infirmary and the Maternity Home, Plaistow. She holds the L.O.S. certificate.

ST. MONICA'S HOME FOR SICK CHILDREN.—Miss L. Maude Newill, has been appointed matron. She was trained at Nottingham Children's Hospital and Guy's Hospital. She has since been ward sister at Pendlebury Children's Hospital and matron at the Jenny Lind Hospital for Children, Norwich.

UNION INFIRMARY, BARNSLEY.—Miss Agnes Melville has been appointed superintendent nurse. She was trained at the Union Infirmary, Rochdale, where she was afterwards charge nurse and night superintendent.

### Presentations.

BELVIDERE HOSPITAL, GLASGOW.—The Christmas gift by the nurses of Belvidere Hospital, Glasgow, to their matron, Mrs. Sinclair, was a handsome reading chair. The inscription on it reads, "Presented to Mrs. Sinclair as a token of love and respect from her Nurses. Christmas, 1902."

BLACKHEATH NURSING INSTITUTE.—Miss Duncan, Principal of the Blackheath Nursing Institute, was presented at Christmas with an extremely handsome brass blotting book and stationery case.

DISTRICT INFIRMARY, ASHTON-UNDER-LYNE.—Miss Alice Chapman, who for a period of nine years has acted as matron of the District Infirmary and Children's Hospital, Ashton-under-Lyne, recently resigned her post. During her tenure of office many improvements, both as regards the nursing and the system of housekeeping, have been effected, and by her kind yet firm and sympathetic treatment of those under her charge Miss Chapman has endeared herself to a large circle. To mark their appreciation of her worth and the benefits they have received at her hands, the present nursing staff and a goodly number of former nurses held a soirée at the infirmary on December 18th, at which Miss Chapman was presented on behalf of the nurses, the house surgeon, and the secretary with a very handsome silver salver and tea service. The presentation was made by Dr. Judson, the house surgeon, who referred in a few well-chosen remarks to the excellent qualities and high capabilities of the retiring matron, whose departure would be so much regretted. Afterwards a programme of songs and dances was gone through, and a most enjoyable evening spent.

### Echoes from the Outside World.

### The Queen's Christmas Dinner.

THE entertainment provided on Saturday by Queen Alexandra for the widows and orphans of the soldiers and sailors who died in the South African war was a splendid success. The invitation from her Majesty had been sent out to 668 widows and 877 children, and in all save 70 instances it had been gladly accepted. To these absentees small hampers containing some of the good things from the feast were despatched at the end of the evening. When the guests entered the house in the City Road known as the Alexandra Trust they found the hall had been converted into a bower of palms, the three dining-rooms were gay with flags, and the tables with their white cloths adorned with vases of flowers. On either side of the hall there was an office for the exchange into money of the vouchers supplied to the guests for the payment of their travelling expenses. Several of the guests were too small to walk, and were carried in their mothers' arms. But there were always willing helpers anxious to take the babies whilst the mothers enjoyed their meal. At the right hand of each guest was a bottle of Schweppe, a roll of bread, a couple of crackers (with several stacks of reserves at irregular intervals), a tasteful New Year's card, and a box of biscuits given by Messrs. Guillot. Added to these each visitor received chocolate from Messrs. Rowntree, a number of the *Graphic* from the proprietors of the paper, and each child a toy from Mr. Whiteley. The ménu of the dinner was given in this column last week. When the feasting was finished Sir Thomas Lipton called for silence and delivered the Queen's message, which ran as follows: "Pray convey the expression of my very best wishes to all my guests in the Alexandra Trust. May they spend a very happy day, and may God help and bless them throughout the coming year." (Signed) "The Queen." This was read out on each floor and a message of thanks was sent to her Majesty by Sir Thomas from all present. An entertainment of two hours completed the day's pleasure, and those who took the organisation in hand had the proud happiness of knowing that there had been no hitch to mar the proceedings, and that the guests left the building with a feeling of satisfaction they are never likely to lose.

### The Coronation Durbar.

THIS week will be memorable in the annals of Indian history. The imposing ceremonies connected with the Coronation Durbar commenced on Monday at Delhi, when the Viceroy made his State entrance into the city. The procession from the railway station through the principal thoroughfares to the great plains beyond was one of surpassing magnificence. The Viceroy and Lady Curzon and the Duke and Duchess of Connaught, mounted on gigantic elephants, were preceded by detachments of troops, and followed by a long line of the native princes and chiefs, also on elephants, with gorgeous trappings and howdahs. Then came almost endless carriages containing the official hierarchy, detachments of cavalry, and, lastly, a hundred and fifty more elephants, bearing the retinues of the native princes. On Tuesday the Viceroy opened an Exhibition of Indian Art. The Durbar itself was held on Wednesday.

### Mr. Chamberlain in Natal.

THE Colonial Secretary and Mrs. Chamberlain arrived at Durban on Boxing Day, and were received on the landing stage by the Governor of Natal and Lady McCullum, the Premier and others. After numerous introductions had been made, the whole party drove to the Town Hall amid much enthusiasm, which culminated when the visitors entered the densely thronged building. An address of welcome having been read, and Mr. Chamberlain having come forward to speak in reply, the entire audience rose and cheered till the roof rang again. The essence of the speech was the description by the right hon. gentleman of his mission, which he said were first to express on behalf of the Mother-country sympathy with her kinsmen over seas, a desire to understand them better, and the hope of more personal and closer relations; and secondly, to gain information which he could nowhere obtain so successfully or so fully as on the spot. From the Town Hall Mr. Chamberlain, his wife and party, drove to the Marine Hotel, where a public luncheon in their honour was given. Here, in response to the toast of his health, he made a longer speech, in the course of which he bore eloquent testimony to the great services rendered to South Africa by Lord Milner. Mr. Chamberlain referred to the Boers as brave antagonists who need feel no humiliation at their defeat; said that nothing should be done to revive old animosities; and declared that we must give the Boers equality, and frankly offer them our hand. Self-government for the new colonies would, he hoped, come soon, but no greater mistake could be made than to hasten it prematurely. After luncheon on Sunday Mr. and Mrs. Chamberlain left Durban for Pietermaritzburg, arriving at the capital at six o'clock in the evening, receiving a splendid welcome. On Monday the Colonial Secretary was presented with an address of welcome, and made a short speech in reply, in the course of which he expressed the hope that the present visit would form a precedent for a periodical tour of a like nature by the representative of the Colonies in the Cabinet. On Tuesday he proceeded to Colenso.

### The late Archbishop of Canterbury.

ON Saturday the mortal remains of Frederick Temple, Primate of All England, were laid to rest in Canterbury Cathedral. Dr. Temple died, as he desired, in harness. Though born 81 years ago on the 30th of last November, his life from the outset, when the death of his father, an officer in the Army, left him without financial resources, to the day when, in spite of failing powers, he delivered his speech in the House of Lords on the Education Act, was one of strenuous endeavour. As head-master of Rugby, as Bishop of Exeter, as Bishop of London, and finally as Archbishop of Canterbury, he worked with unremitting energy to promote the interests he had at heart. No one who was present at the Coronation, and least of all the King and Queen, who so constantly during his last illness made special inquiries concerning him, will forget the sudden and pathetic weakness which he displayed in Westminster Abbey on that memorable occasion. It was probably the beginning of the end, but Dr. Temple, of whom Lord Salisbury used to say "he was ashamed to talk of hard work before the Archbishop," refused to give up any of his duties, and he set an example for all time of faithful service in whatever capacity he filled.

### Letters at Christmas.

IN many quarters it is being recognised that something must be done to relieve the Post Office of the dreadful pressure put upon it at Christmas time under existing circumstances. At Manchester last week special pillar boxes were reserved for letters intended for delivery only in the city and suburbs. Far more important was a movement tried at Rochdale. The inhabitants were requested to post their Christmas letters in advance, between the 17th and 22nd, under an official guarantee that they should not be delivered before the 25th. They were distinguished from any other letters by a special mark, and were, upon receipt, sorted at once, tied up in bags and left ready for delivery on Christmas morning. They reached their recipients before 10 A.M. on the 25th.

### For Reading to the Sick.

THE OLD-YEAR'S BLESSING.

I HAVE faded from you,
But one draweth near,
Called the Angel-guardian
Of the coming Year.

If my Gifts and Graces
Coldly you forget,
Let the New-Year's Angel
Bless and crown them yet.

For we work together;
He and I are one:
Let him end and perfect
All I leave undone.

I brought Joy to brighten
Many happy days;
Let the New-Year's Angel
Turn it into Praise.

If I gave you Sickness,
If I brought you Care,
Let him make one Patience,
And the other Prayer.

. . . . .

May you hold this Angel
Dearer than the last—
So I bless his future,
While he crowns my past.

*A. Proctor*

To-day is the foreground of all the yesterdays that have ever been, each hour is the foreground of every to-morrow which Eternity holds.—*Una A. Taylor.*

May it be our blessedness, as years go on, to add one grace to another and advance upward, step by step, neither neglecting the lower after attaining the higher, nor aiming at the higher before attaining the lower. The first grace is faith, the last is love; first comes zeal, afterwards comes loving kindness; first comes humiliation, then comes peace; first comes diligence, then comes resignation. May we learn to mature all graces in us;—fearing and trembling, watching and repenting, because Christ is coming; joyful, thankful, and careless of the future, because He is come.

*J. H. Newman.*

If we have wandered, if we have turned a deaf ear to the Shepherd's voice, let us return, let us go to our Lord right humbly. See! He calls us to Him; He gives us another chance; He says, "Begin again."—*H. J. W. Buxton.*

I do not ask, O Lord, that Thou shouldst shed
Full radiance here;
Give but a ray of peace, that I may tread
Without a fear.
I do not ask my cross to understand,
My way to see—
Better in darkness just to feel Thy Hand,
And follow Thee.
Joy is like restless day, but peace divine
Like quiet night:
Lead me, O Lord, till perfect day shall shine
Through Peace to Light.

*Adelaide Proctor.*

### Notes and Queries.

**The Editor is always willing to answer in this column, without any fee, all reasonable questions, as soon as possible.**

**But the following rules must be carefully observed:—**

**1. Every communication must be accompanied by the name and address of the writer.**

**2. The question must always bear upon nursing, directly or indirectly.**

**If an answer is required by letter a fee of half-a-crown must be enclosed with the note containing the inquiry, and we cannot undertake to forward letters addressed to correspondents making inquiries. It is therefore requested that our readers will not enclose either a stamp or a stamped envelope.**

*South Africa.*

(104) Will you kindly give me the names and addresses of nursing institutions in Capetown, Port Elizabeth, and Johannesburg? Are there openings for trained masseuses in South Africa?—*E. M. F.*

Have you not read our repeated warnings to nurses that the supply of nurses in South Africa far exceeds the demand? The lady superintendents of the nursing associations both at Capetown and Johannesburg have written to us stating that the applications from nurses are so numerous that it is impossible to even reply to them.

*Persehouse Pensions.*

(105) "Mr. Parkes bequeathed his residuary estate of about £50,000, provided he had no children, to found 'Persehouse Pensions' for aged and distressed persons of the middle and upper classes in the counties of Staff rd and Worcester." Can you kindly tell me if the above charity has been founded and where I can obtain particulars?—*H. P.*

There is no record of such a charity in our possession; also you give no date nor authority for your quotation, so that we cannot trace its origin.

*Address.*

(106) Will you kindly give me the address of Mrs. K., midwife who takes pupils, and has a large district in the south-east end of London in connection with the York Road Lying-in-Hospital, mentioned in "Three Months' Midwifery Training" in THE HOSPITAL some weeks ago—*Nurse A.*

We cannot give private addresses.

Will you kindly tell me the address of the Marchioness of Dufferin, with whom I wish to communicate on the subject of maternity work in India?—*V. K. C.*

We cannot give private addresses, but if your work is in connection with the medical work carried on by the charities bearing the name of Lady Dufferin, the Secretary, the National Association for Supplying Female Medical Aid to the Women of India, 9 Clarence Place, Belfast, would be able to do so.

*Coached for Exams.*

(107) Can you tell me if it is possible for a nurse to be coached for her exams. either by correspondence or otherwise at a reasonable fee?—*E. M.*

The Secretary, the Trained Nurses' Club, 12 Buckingham Street, Strand, W.C., could doubtless arrange this for you.

*Home.*

(108) Can you kindly tell me of a home or hospital where a woman of 21, paralysed from the waist downwards, could be admitted free of charge?—*Nurse F.*

Not as a permanency. If there is any hope of cure she might be admitted for treatment at one of the special hospitals for this class of disorder.

Can you kindly tell me of a home where an old lady who suffers much from bronchitis during the winter could be received for 7s. 6d. per week?—*Aetas.*

All Saints' Convalescent Home, 8 Pevensey Road, St. Leonards, might be willing to receive her.

**Standard Books of Reference.**

**"The Nursing Profession: How and Where to Train." 2s. net; post free 2s. 4d.**

**"Burdett's Official Nursing Directory." 3s. net; post free, 3s. 4d.**

**"Burdett's Hospitals and Charities." 5s.**

**"Hospital Expenditure: The Commissariat." 2s. 6d.**

**"The Nurses' Dictionary of Medical Terms." Cloth, 2s.; leather, 2s. 6d. net.; post free, 2s. 8d.**

**"Burdett's Series of Nursing Text-Books." 1s. each.**

**"A Handbook for Nurses." (Illustrated). 5s.**

**"The Physiological Feeding of Infants." 1s.**

**"The Physiological Nursery Chart." 1s.; post free, 1s. 3d.**

**All these are published by the SCIENTIFIC PRESS, Ltd., and may be obtained through any bookseller or direct from the publishers, 28 and 29 Southampton Street, London, W.C.**

